# The Neural Signatures of Processing Semantic End Values in Automatic Number Comparisons

**DOI:** 10.3389/fnhum.2015.00645

**Published:** 2015-11-27

**Authors:** Michal Pinhas, Chananel Buchman, Dmitri Lavro, David Mesika, Joseph Tzelgov, Andrea Berger

**Affiliations:** ^1^Department of Psychology, Zlotowski Center for Neuroscience, Ben-Gurion University of the NegevBeer Sheva, Israel; ^2^Department of Behavioral Sciences, Ariel UniversityAriel, Israel; ^3^Department of Brain and Cognitive Sciences, Ben-Gurion University of the NegevBeer Sheva, Israel

**Keywords:** end effect, automatic numerical processing, event-related potential (ERP), P3, size congruity effect, precuneus

## Abstract

The brain activity associated with processing numerical end values has received limited research attention. The present study explored the neural correlates associated with processing semantic end values under conditions of automatic number processing. Event-related potentials (ERPs) were recorded while participants performed the numerical Stroop task, in which they were asked to compare the physical size of pairs of numbers, while ignoring their numerical values. The smallest end value in the set, which is a task irrelevant factor, was manipulated between participant groups. We focused on the processing of the lower end values of 0 and 1 because these numbers were found to be automatically tagged as the “smallest.” Behavioral results showed that the size congruity effect was modulated by the presence of the smallest end value in the pair. ERP data revealed a spatially extended centro-parieto-occipital P3 that was enhanced for congruent versus incongruent trials. Importantly, over centro-parietal sites, the P3 congruity effect (congruent minus incongruent) was larger for pairs containing the smallest end value than for pairs containing non-smallest values. These differences in the congruency effect were localized to the precuneus. The presence of an end value within the pair also modulated P3 latency. Our results provide the first neural evidence for the encoding of numerical end values. They further demonstrate that the use of end values as anchors is a primary aspect of processing symbolic numerical information.

## Introduction

When we are asked to compare the values of number pairs, response latencies usually decrease as the intrapair distance between the numbers increases. For example, responses are faster for comparing 2 with 7 (i.e., a distance of 5) than for comparing 3 with 4 (i.e., a distance of 1). This response pattern is known as the distance effect (Moyer and Landauer, 1967). Together with other behavioral and neural signatures it suggests a compressed scaling of magnitudes (e.g., Dehaene, 2003; Feigenson et al., 2004; Verguts and Fias, 2004), often metaphorically referred to as the “mental number line." Another factor that can affect performance in the number comparison task is whether the pair contains an end value (i.e., the smallest/largest number). The end effect is characterized by a faster response when comparing pairs involving end values relative to pairs involving values from the middle of a given number range (e.g., Banks, 1977; Leth-Steensen and Marley, 2000). For comparisons involving ordered sets of non-numerical items, end effects are generally quite robust for pairs containing items located at either end of the ordering. For comparisons of numerical stimuli, this effect tends to be stronger for lower (e.g., 1 or 2) rather than upper end values (e.g., 8 or 9) due to the integrated influence of the size effect, which consists of faster comparative responses for smaller numbers than for larger numbers (Moyer and Landauer, 1967; Banks et al., 1976). The end effect is explained by assuming that participants learn the given number range early on in the experiment (even if it is not explicitly specified) and accordingly use information regarding which values are at the ends of that range to help guide their comparative judgments (Schwarz and Stein, 1998). Moreover, a recent study showed that participants respond faster to comparisons of the same number pairs under a smaller versus larger number range, which is consistent with the idea that the knowledge acquired throughout the experiment about the given number range adjusts the proximity of the same numbers along the “mental number line" (Pinhas et al., 2013).

Leth-Steensen and Marley (2000) proposed a model to account for several effects that characterize intentional comparisons made on a fixed, ordered set of symbolic stimuli. Their model assumes that reaching a decision in such a case is based on two independent processes that run in parallel, racing against each other. The first process is an analog comparison process that encodes which number in the pair is numerically larger/smaller and evokes the distance effect. The second process is an end anchor identification process, which is carried out to determine if the pair contains an end value. When one – or both – members of the pair are mapped as an end value, the output of the end-anchor identification process signifies that this number is the smaller/larger in the pair (e.g., Potts, 1974; Banks, 1977). The activation from the end anchor module is minimal for pairs that do not include end values, and therefore the decision is based on the analog comparison process. In contrast, if the pair includes an end value, the contribution from the processing of the intrapair distance turns out to be fairly minimal, and the decision is based on the end anchor identification process. However, as pointed out by Leth-Steensen and Marley (2000), distance effects may still occur in pairs that involve end values if the analog comparison process finishes before the end anchor identification process. In fact, there are certain cases in which distance effects for end-value pairs are predicted by the model (for further discussion of this point see Leth-Steensen and Marley, 2000).

Behavioral evidence supporting the involvement of both processes proposed by Leth-Steensen and Marley (2000) is also found in the case of automatic number comparisons, that is, when number values are processed even when determining the value is not part of the task requirement (e.g., Bargh, 1992; Tzelgov, 1997; Tzelgov and Ganor-Stern, 2005). The advantage of studying automatic numerical comparisons is that under such conditions, the processing is less contaminated by the specific task demands, allowing us to get an in-depth view of the underlying mental representations. For example, consider the physical comparison task (also known as the numerical Stroop task) in which participants are presented with a pair of digits that differ both in their font size and numerical values. Participants are asked to choose the digit that is physically larger. Although the processing of the number values under such conditions is not beneficial for the task and participants are instructed to ignore these values, they usually do not succeed in doing so. Hence, responses are faster and more accurate for congruent trials (e.g., 3 vs. 5), where the physically larger digit is also greater in value, than for incongruent trials (e.g., 3 vs. 5), where the physically larger digit is smaller in value (Henik and Tzelgov, 1982). The reaction time (RT) difference between these two conditions (i.e., incongruent minus congruent) is referred to as the size congruity effect (SiCE). The SiCE has been frequently used as a marker for the automatic processing of numbers and was found to be enlarged for pairs with larger compared to smaller intrapair distance (e.g., Henik and Tzelgov, 1982; Tzelgov et al., 1992; Pansky and Algom, 1999; Schwarz and Ischebeck, 2003; Cohen Kadosh et al., 2007; Parnes et al., 2012; Pinhas and Tzelgov, 2012).

Recently Pinhas and Tzelgov (2012) argued that the two processes hypothesized by Leth-Steensen and Marley (2000) determine the processing speed of the task-irrelevant number values in the physical size comparison task. The analog comparison process results in an increase in the SiCE with the increase in the intrapair distance, and the end-anchor identification process leads to faster automatic processing of the relative numerical magnitudes for pairs that contain end values than for pairs that contain non-end values, resulting in a larger SiCE for the former type of pairs (for a possible model see Schwarz and Ischebeck, 2003). To support these claims, Pinhas and Tzelgov (2012) tested modulation of the SiCE by two task-irrelevant factors: the numerical intrapair distance and the presence of the smallest end value in the range within the pair. They manipulated the stimulus set such that for different groups of participants 0, 1, or 2 were used as the smallest numbers in terms of their absolute values.^1^ They found that pairs containing the smallest number 0 or 1 resulted in (a) an enlarged SiCE that in most cases was (b) minimally modulated by the intrapair distance. This pattern of results was termed the *automatic end effect*. In contrast, results obtained for pairs containing the number 2 when it was the smallest value in the set resembled those obtained for pairs containing non-end numbers and did not produce the automatic end effect. The SiCE in these latter cases was smaller overall and showed the classical effect of modulation by intrapair distance. Thus, Pinhas and Tzelgov (2012) found behavioral evidence supporting the involvement of both the analog comparison process and the end-anchor identification process in the case of automatic processing of number values as instantiated by different modulations of the SiCE.

The findings of an automatic end effect in physical comparisons of pairs containing the smallest number 0 or 1, but not for pairs containing the smallest number 2, led Pinhas and Tzelgov (2012) to conclude that only numbers that are encoded in long-term semantic memory as “the smallest” (such as 0 or 1) elicit the end effect when number values are processed, although their processing is task-irrelevant. In other words, the end effect in the case of physical size comparisons does not seem to reflect the episodic serial position of a number at a given experimental range, as happens with the end effect in numerical comparisons where number values are intentionally compared to one another. Rather, it seems that for a number to be automatically perceived as “the smallest” it has to be *semantically* encoded as such. Accordingly, in daily life we usually experience either 0 or 1 (in the absence of 0) as being the smallest number/the first number: 1 is the most frequently used positive integer that we start counting from and learn first when we encounter symbolic numbers, and 0 is the smallest non-negative integer that we formally acquire in early childhood (typically only after children learn the meaning of the first counted number words, i.e., including 1; e.g., Wellman and Miller, 1986) and use more in mathematical or abstract thinking contexts. In contrast, although 2 is a relatively small number, in life we are not usually exposed to situations in which it is “the smallest,” and thus it is not perceived this way under conditions of automatic processing, even if it is indeed used as the smallest end value in the set (Pinhas and Tzelgov, 2012). The similar response patterns showing an automatic end effect for pairs containing the smallest number 0 or 1 cannot be explained on the basis of familiarity because these numbers do not share the same level of familiarity. Whereas 1 is by far the most frequent number, the frequency of 0 is much lower. In fact, the frequency of 0 is lower than that of the numerals 1–9 and the decades 10–90 (Dehaene and Mehler, 1992). Hence, given these considerations and based on our earlier findings (Pinhas and Tzelgov, 2012), we are claiming that the automatic end effect observed in the physical comparison task can serve as a marker dissociating between numbers that are *semantically* tagged as end values (i.e., 0 and 1) and numbers that are not tagged as such (e.g., 2).

Previous event-related potential (ERP) studies with symbolic numerosities (e.g., digits, written number words) have used both the numerical comparison and the physical comparison tasks to examine the neural processing associated with the distance and size congruity effects. Modulations of ERPs by numerical distance typically emerge over parietal sites and occur at both early and/or later time windows in a variety of tasks (e.g., Dehaene, 1996; Temple and Posner, 1998; Libertus et al., 2007; Szűcs and Soltész, 2007; Szűcs et al., 2007; Hyde and Spelke, 2009). For example, early voltage differences (<250 ms) as a function of intrapair distance were found in the numerical comparison task over parieto-occipital sites mostly for the P2p (second posterior positivity) component and in some cases also slightly earlier in the waveform for the N1 component or in the transition between the N1 and the P2p (e.g., Dehaene, 1996; Temple and Posner, 1998; Libertus et al., 2007). The N1 is an early sensory component that is evoked by any visual stimuli. Extensive evidence further suggests that the N1 reflects a mechanism of attention allocation (e.g., Hillyard et al., 1990; Luck, 2005). Temple and Posner (1998) suggested that an earlier distance effect that is already apparent in the first negativity might be related to greater attention allocation in cases where the task is less practiced. Distance effects on the N1 amplitude with non-symbolic numbers (e.g., dot arrays) were also attributed to attentive object tracking processes found for small (but not large) numbers (Hyde and Spelke, 2009) and to differences in sensory processing rather than to distinct number-related processing (Libertus et al., 2007).

The P2p ERP distance effect is obtained in numerical comparisons of both symbolic and non-symbolic number formats in adults as well as in children, and thus it was suggested to reflect the neural activation of an abstract and notation-independent number processing mechanism that is already present early in development (e.g., Dehaene, 1996; Temple and Posner, 1998; Libertus et al., 2007; Hyde and Spelke, 2009, 2012). Neuroimaging studies associate the brain locus of this number system with the parietal cortex, specifically with the horizontal segment of the intraparietal sulcus (IPS). This area is found to be modulated by the distance between numerals (e.g., Pinel et al., 2001; Dehaene, 2003; Feigenson et al., 2004; Ansari et al., 2005; Kaufmann et al., 2005; Hyde and Spelke, 2012), as well as by the congruity relations between numerical and physical size dimensions (Dehaene et al., 2004; Cohen Kadosh et al., 2007). Similar ERP distance effects were also reported in the physical comparison task – where the processing of the number values was task-irrelevant – between 140 and 320 ms poststimulus (Szűcs et al., 2007; Szűcs and Soltész, 2007).

Studies that have tested the ERPs associated with the SiCE in the physical comparison task reported that the effect is expressed in the P3 (known as the P300 or P3b) component. The P3 is distributed over centro-parietal electrodes, with decreased amplitude and delayed latency for the incongruent compared to the congruent condition. Furthermore, the P3 negatively correlates with RT such that faster responses (typically for congruent trials) are associated with increased P3 amplitudes (e.g., Schwarz and Heinze, 1998; Cohen Kadosh et al., 2007; Szűcs and Soltész, 2007; Szűcs et al., 2007, 2009; Parnes et al., 2012). More generally, the P3 is considered to reflect stimulus categorization and evaluation processes that vary between tasks. P3 amplitude was shown to be sensitive to target probability and P3 latency was linked to the amount of time required to evaluate a stimulus (e.g., Kutas et al., 1977; McCarthy and Donchin, 1981; Kok, 2001; Polich, 2007, 2012). Furthermore, P3 congruency-related effects were considered to be evidence for conflict at the stimulus level, although both stimulus and response conflicts were found to contribute to the SiCE (Schwarz and Heinze, 1998; Cohen Kadosh et al., 2007; Szűcs and Soltész, 2007; Szűcs et al., 2007, 2009).

The goals of the present study were twofold. The first goal was to explore the electrophysiological correlates associated with processing semantic end values under conditions of automatic number comparisons and distinguish them from those associated with numerical-distance-based processing. The second goal was to explore the neural generators of processing semantic end values. For these purposes, ERPs were recorded for two groups of adult participants who were asked to physically compare pairs of digits. We manipulated the task-irrelevant factor of the smallest number in the set so that for one group of participants it was 0 and for the other it was 1. We focused on the processing of lower end values for several reasons. As mentioned, we recently demonstrated that only numbers that are semantically encoded as “the smallest” (i.e., 0 and 1, but not 2) produce end effects when number values are processed automatically (Pinhas and Tzelgov, 2012). Furthermore, prior work shows that the minus sign is ignored when negative numbers are physically compared, so that numbers are processed automatically in terms of their absolute and not real values (Tzelgov et al., 2009; Pinhas and Tzelgov, 2012). It follows that 0 serves as the lowest end value that can be perceived as such when performing physical comparisons. In contrast, there is no definitive “upper limit” for larger number values because numbers do not really end. Consequently, potential upper end values within the single-digit number range are presumably not expected to be semantically tagged as the “largest.” In fact, recent behavioral work from our lab confirmed that prediction, revealing that pairs that contained the largest number within the single-digit range (e.g., 8 or 9) did not produce automatic end effects (Goldman and Tzelgov, in preparation).

Given that electrophysiologically the SiCE is reflected in the P3 component and that behaviorally the effect is enlarged for pairs that include semantic end values, we expected that the end-anchor identification process would be expressed in increased P3 congruity effect (incongruent minus congruent) amplitudes for pairs that contain the smallest end value in the set (termed “smallest” pair type) compared to pairs that contain non-end numbers (termed “non-smallest” pair type). Similar to previous studies, we also manipulated the irrelevant numerical intrapair distance. We expected that an analog comparison process would be reflected by an early distance ERP effect that would be apparent for the P2p component and possibly also for the earlier N1 component (e.g., Dehaene, 1996; Temple and Posner, 1998; Libertus et al., 2007; Szűcs and Soltész, 2007; Szűcs et al., 2007).

## Materials and Methods

### Participants

Thirty-two undergraduate students from Ben-Gurion University of the Negev participated in the experiment for payment. All were native Hebrew speakers with no history of neurological illness and all had normal or corrected-to-normal vision. Participants were randomly assigned to one of the two experimental groups. Data of four participants were discarded from the final analysis due to insufficient artifact-free correct trials for signal averaging. The average age of the remaining 28 participants was 24.54 years (*SD* = 3.05, all right-handed, 19 females). This research was approved by the Helsinki Committee of the Ministry of Health. All participants gave written informed consent prior to the beginning of the experiment.

### Apparatus and Stimuli

The experiment was conducted on a personal computer running Windows XP with a 15-inch monitor. E-Prime v1.2 software controlled the presentation of stimuli (Schneider et al., 2002). Participants responded by pressing the two extreme outer keys (out of four aligned keys) on the E-prime serial response box with their left and right index fingers.

The stimuli were generated from single-digit integers (from 0 to 9). Number pairs were designed to fit close (i.e., an average of the intrapair distances of 1 and 2) and far (i.e., an average of the intrapair distances of 4 and 6) distances, in four pair types: “0 pairs” (0 paired with 1, 2, 4, and 6), “1 pairs” (1 paired with 2, 3, 5, and 7), “2 pairs” (2 paired with 3, 4, 6, and 8), and “3 pairs” (3 paired with 4, 5, 7, and 9). This created a total of 32 pairs, given that each number appeared once on the left and once on the right side of the computer screen. Two sets of stimuli were created out of the 32 pairs: one for the congruent condition, where the numerically larger number was also physically larger, and one for the incongruent condition, where the numerically larger number was physically smaller. Each pair was repeated 30 times in each condition for a total of 1,920 pairs. All pairs were presented for the group of participants in which 0 was used as the smallest number (i.e., 16 pairs × 2 left/right side × 2 congruencies × 30 repetitions = 1,920). However, 0 pairs were excluded for the group of participants in which 1 was used as the smallest number, resulting in a total of 1,440 pairs (i.e., 12 pairs × 2 left/right side × 2 congruencies × 30 repetitions = 1,440). Thus, 2 pairs and 3 pairs were presented for both groups and were always used as non-end pairs. Pairs containing the smallest end value in the set were termed “smallest” pair type (i.e., 0 pairs/1 pairs for the group in which 0/1 was used as the smallest number, respectively). Pairs that did not contain the smallest end value in the set were termed “non-smallest” pair type (i.e., 1 pairs, 2 pairs, and 3 pairs/2 pairs and 3 pairs for the group in which 0/1 was used as the smallest number, respectively). **Supplementary Table S1** lists all pairs used in the experiment.

The distance between the two presented numbers was 1.15°, assuming a viewing distance of 60 cm. Numbers appeared in “Courier New” bold font that was colored white on a black background on both sides of the center of the screen in either small (about 0.9° high and 0.6° wide) or large (about 1.05° high and 0.8° wide) sizes.

### Procedure

The task was always a physical comparison. Participants were randomly assigned to one of two groups, which differed according to the smallest number that was presented to them: 0 or 1. The participants sat on a chair in front of the monitor with their index fingers on the response keys. Each trial started with the presentation of a fixation dot at the center of the screen for 250 ms, followed by a blank screen randomly presented for 200, 250, or 300 ms, followed by a pair of numbers that remained visible until a response was made. After response, a blank screen was randomly presented for 500, 600, or 700 ms before the next trial started. Because the pair of numbers remained visible on the screen until response the duration of the visual stimulus presented differed between trials. In turn, these duration differences are expected to result in differences in the latency of the visually evoked potentials for target offset. However, these potentials would likely be occurring only after ∼450 ms poststimulus, that is, later than the time windows used in the ERP analyses (for details see EEG Recording and Analysis).

Participants were instructed to ignore the numerical values and to select the number that appeared physically larger on the screen as quickly and as accurately as possible. They were asked to press the outer left/right key on the response box if they chose the number on the left/right, respectively. A short training session was presented first, followed by 32/24 successive blocks of 60 trials each for the group in which 0/1 was used as the smallest number, respectively. Feedback of accuracy percentage and mean RT of the correct responses was provided at the end of each block. A self-paced break was presented after each block. During training a short error beep was sounded in cases where an incorrect response was made. Trials were randomly ordered.

### Behavioral Analysis

Reaction times shorter than 100 ms (six trials) were excluded from all analyses. Error rates were quite low (averaged 2.9%, ranging from 1 to 7%) and preliminary analysis suggested no speed-accuracy trade-offs across subjects. Mean RTs of correct responses were submitted to a four-way repeated measures analysis of variance (ANOVA) with *the smallest number* (0, 1) as a between-participants variable and *congruency* (congruent, incongruent), *pair type* (smallest, non-smallest), and *distance* (close, far) as within-participants variables. Furthermore, a separate repeated-measures ANOVA was conducted for each smallest number group with *congruency* (congruent, incongruent) and *pair type* (0/1/2/3 pairs for the group in which 0 served as the smallest number; 1/2/3 pairs for the group in which 1 served as the smallest number) as within-participants variables. The significance level was defined as *p* < 0.05.

### EEG Recording and Analysis

The ongoing EEG was recorded in a sound-attenuated, electrically shielded room from 128 scalp sites using the EGI Geodesic Sensor net and system (Tucker, 1993). Four anterior channel pairs were used to detect eye blinks, and an additional anterior-lateral channel pair was used to detect eye movements. Recordings were referenced online to the VREF (Cz) channel, band-pass filtered at 0.1–100 Hz and digitized with a 250 Hz sampling rate using a 16-bit A/D converter. Electrode impedances were maintained below 40 kΩ, an acceptable level for this system (Ferree et al., 2001).

Oﬄine the data were processed in Net Station 4.4.1 (Electrical Geodesics, Oregon, OR, USA). A 40 Hz digital low-pass filter was first applied to the data. Then the same trials as included in the behavioral analysis were segmented into epochs starting at 200 ms before the onset of the number pair to 600 ms poststimulus. The resulting epochs were subjected to an automated artifact and bad channels detection procedure. The artifact parameters used in this procedure were adjusted and verified visually by looking at ∼20% randomly selected epochs of each of the individual data sets. Artifacts were identified based on the following parameters: (a) ocular artifacts - a max–min amplitude difference higher than 115 μV from eye blink channel pairs and a max-min amplitude difference higher than 85 μV from an eye movement channel pair, and (b) bad channels – each channel with a max–min amplitude difference higher than 140 μV. Epochs detected as containing ocular artifacts and/or those with 15 or more bad channels were excluded. Artifact-free trials were averaged to experimental conditions of interest (i.e., all eight congruency × pair type × distance combinations) for each participant and baseline corrected to the average amplitude from -200 to 0 ms before stimulus onset. Prior to baselining, all channels were referenced to a PARE-corrected overall average to compensate for the polar average reference effect (PARE; Junghöfer et al., 1999) caused by uneven surface sampling. On average, 167.69 (*SD* = 35.26) trials were retained for each participant per a given congruency × pair type × distance condition. Data from four participants were excluded because they had fewer than 60 usable correct trials for one or more conditions. Accordingly, 28 participants were included in the final analysis: 15 in the “0 as smallest” group and 13 in the “1 as smallest” group. Finally, grand averages and topographical maps across all participants, as well as for the two different “smallest number” groups, were generated.

**FIGURE 1:**
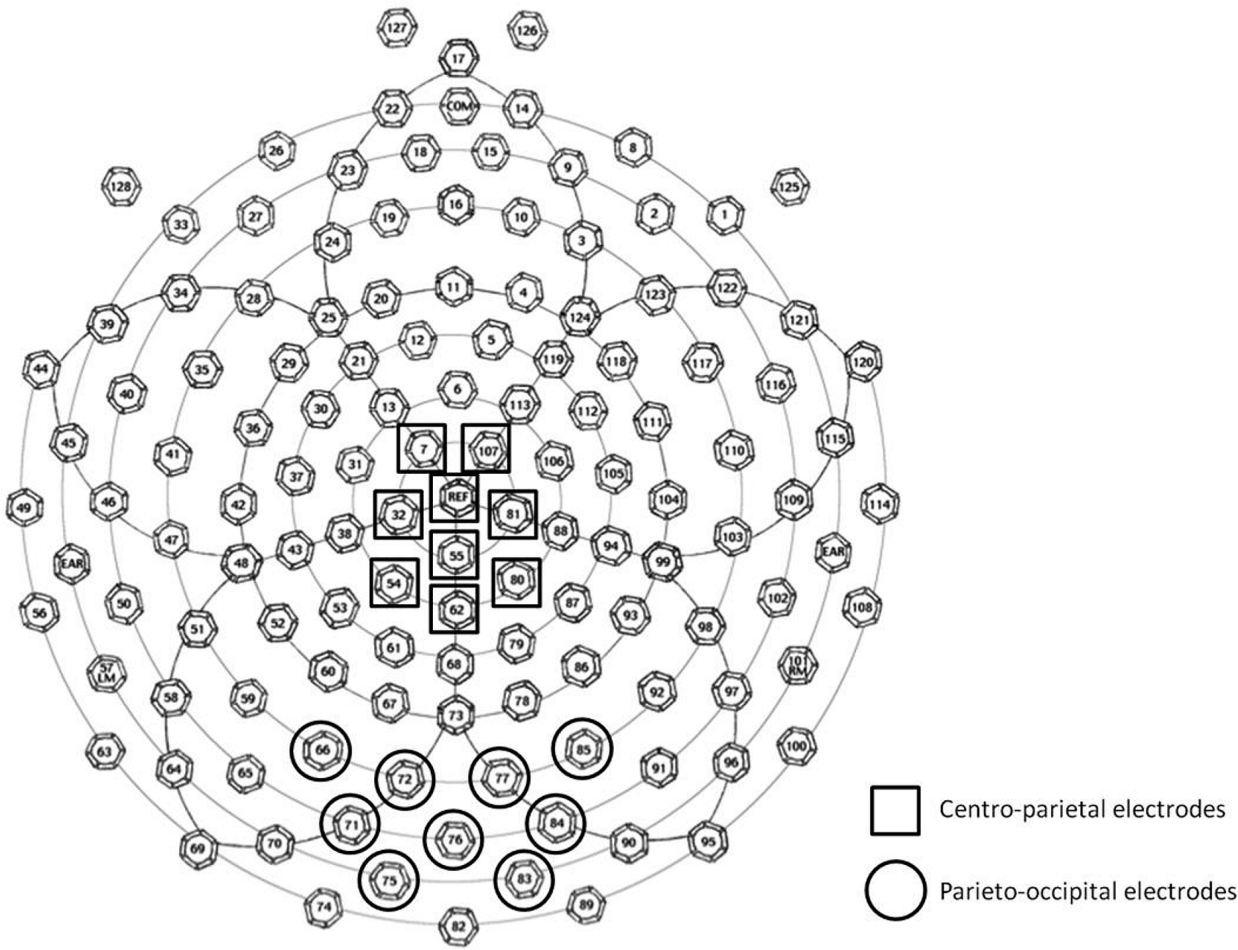
**Schematic drawing of 128-electrode locations on the EGI Geodesic Sensor Net.** The two groups of electrodes used for averaging and analysis are marked.

The selection of ERP components and latencies for analysis was guided by previous research (e.g., Dehaene, 1996; Temple and Posner, 1998; Schwarz and Heinze, 1998; Cohen Kadosh et al., 2007; Libertus et al., 2007; Szűcs and Soltész, 2007; Szűcs et al., 2007, 2009; Hyde and Spelke, 2009, 2012; Parnes et al., 2012) and confirmed by visual inspection of the grand-average waveforms and topographical maps across participants. We identified three time windows corresponding to the three observed ERP components of interest: N1 (covering the first posterior negative-going deflection that follows the P1; 165–200 ms), P2p (covering the second posterior positive-going deflection; 230–280 ms), and P3 (covering an extended positive-going deflection over both centro-parietal and posterior sites; 300–370 ms). Two groups of electrodes were defined for statistical analysis: a parieto-occipital group and a centro-parietal group. The first group included nine central adjacent parieto-occipital electrodes surrounding electrodes O1 and O2 of the 10–20 international standard system. The second centro-parietal group of electrodes included three adjacent midline electrodes from Cz to Pz of the 10–20 international standard system, as well as three immediately adjacent electrodes to the left and right of this midline (see **Figure 1**). As seen in **Figure 1**, these scalp site groupings overlapped with chosen electrode sites from previous ERP studies of number processing using an average reference (N1 and P2p: Dehaene, 1996; Temple and Posner, 1998; Hyde and Spelke, 2009, 2012; P3: Cohen Kadosh et al., 2007; Szűcs et al., 2007, 2009; Parnes et al., 2012).

Visual inspection of the difference waves of the various experimental conditions of interest (e.g., smallest vs. non-smallest pairs; close vs. far) did not reveal differences as a function of pair type over the parieto-occipital group of electrodes. Thus, in order to simplify the data analyses, three-way repeated-measures ANOVAs with the *smallest number* as a between-participants variable and *congruency* and *distance* as within-participants variables were used for testing the mean amplitudes of the N1 and P2p components over the parieto-occipital group of electrodes. For the analysis of the widely distributed P3 component we used both centro-parietal and parieto-occipital electrode groups. The mean amplitudes of the P3 were analyzed using a five-way repeated-measures ANOVA with the *smallest number* as a between-participants variable and *congruency*, *pair type*, *distance*, and *electrodes location* as within-participants variables. An additional analysis testing onset latency (in ms) of the P3 was conducted by analyzing 50% of P3 peak amplitude, spanning from 250 to 500 ms poststimulus, using the same five-way ANOVA design. Compatible follow-up analyses were conducted when needed. Significance was inferred for Greenhouse-Geisser corrected *p*-values < 0.05.

*Post hoc* source localization of the P3 component was performed using the standardized low-resolution brain electromagnetic tomography (sLORETA; Fuchs et al., 2002; Pascual-Marqui, 2002; Jurcak et al., 2007, p. 20), a technique which provides a solution without the need to assume a starting model of dipole locations. The technique extracts current density estimates (sLORETA values) on a space of 6,239 voxels (voxel size: 5 mm × 5 mm × 5 mm), as defined by the digitized MNI152 template. The region of interest (ROI) was determined using a 5,000 iteration bootstrap method that compared between conditions in the P3 mean amplitude analysis time window (i.e., 300–370 ms poststimulus) over the centro-parietal electrodes group. This randomization method performs voxel-by-voxel *t*-tests for dependent measures that are corrected for multiple comparisons. The selected ROI was the centroid voxel that showed maximum difference between conditions in the peak-latency time-window. We then analyzed the sLORETA values to reveal the potential difference in the electric neural generators for the P3 component between conditions of interest.

## Results

### Behavioral Results

**FIGURE 2:**
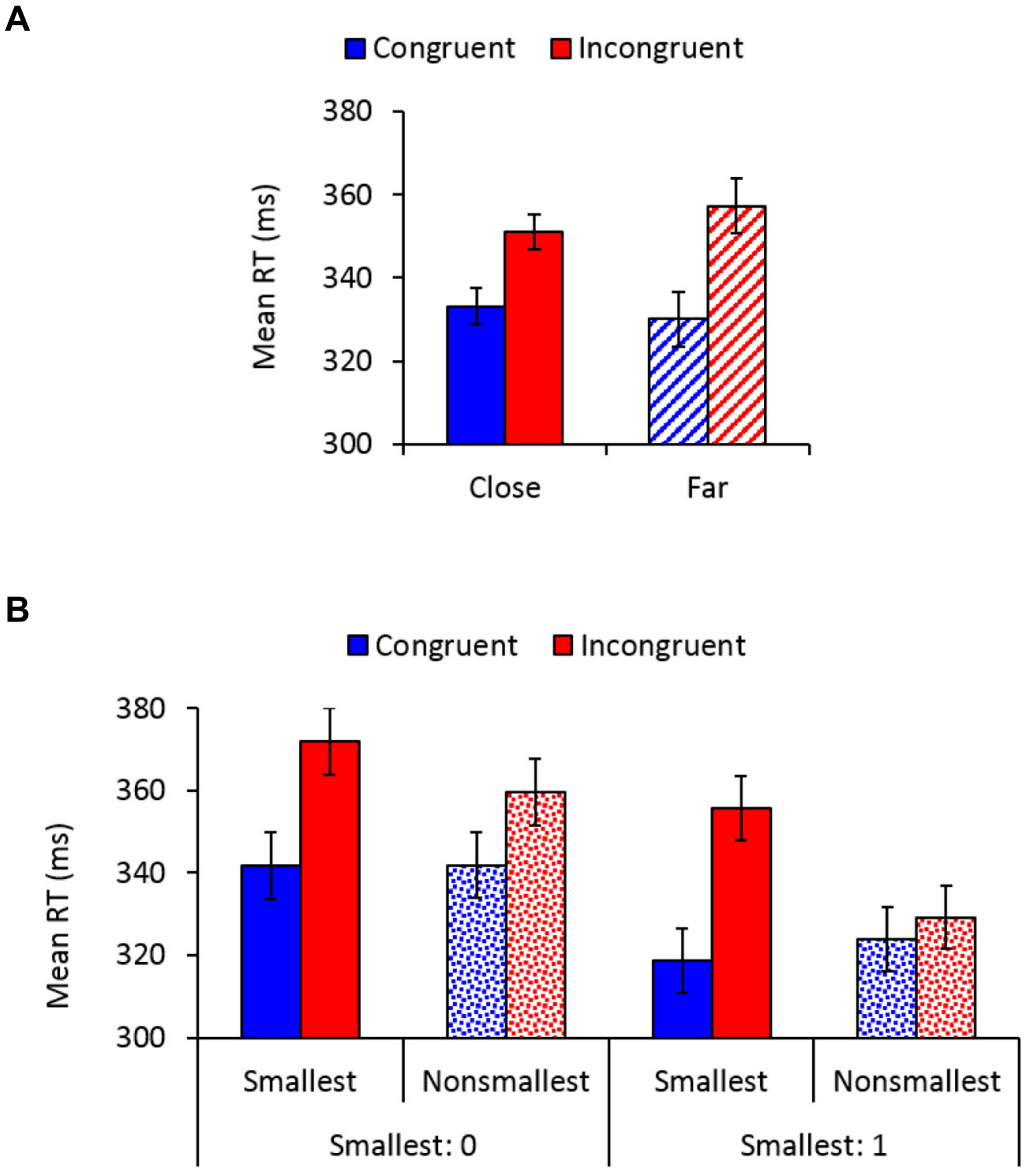
**(A)** Mean reaction times (RTs) as a function of congruency and distance. Error bars denote 0.95 confidence intervals. **(B)** Mean RTs as a function of congruency, pair type, and the smallest number. Error bars denote 0.95 confidence intervals.

Main effects of congruency, *F*_(1,26)_ = 78.08, *MSE* = 363, $\eta_{p}^{2}$ = 0.75, *p* < 0.01, and pair type, *F*_(1,26)_ = 50.54, *MSE* = 77, $\eta_{p}^{2}$ = 0.66, *p* < 0.01, indicated faster responses to congruent (332 ms) versus incongruent (354 ms) trials, and to pairs containing non-smallest numbers (339 ms) versus pairs containing the smallest number (347 ms), respectively. A main effect of distance, *F*_(1,26)_ = 4.52, *MSE* = 29, *p* < 0.05, $\eta_{p}^{2}$ = 0.15, indicated significantly faster responses to close versus far distances, though there was only 2 ms difference between these conditions (342 vs. 344 ms, respectively). As expected, congruency interacted with distance, *F*_(1,26)_ = 28.06, *MSE* = 45, *p* < 0.01, $\eta_{p}^{2}$ = 0.52, so that although the SiCE was significant in both distance conditions [close: *F*_(1,26)_ = 72.01, *MSE* = 122, $\eta_{p}^{2}$ = 0.73; far: *F*_(1,26)_ = 72.86, *MSE* = 285, *p* < 0.01, $\eta_{p}^{2}$ = 0.74], it was larger for physical comparisons of far (27 ms) versus close (18 ms) number values (**Figure 2A**). Importantly, congruency interacted with pair type, *F*_(1,26)_ = 35.57, *MSE* = 190, *p* < 0.01, $\eta_{p}^{2}$ = 0.58, demonstrating a larger SiCE for pairs containing the smallest number [34 ms; *F*_(1,26)_ = 63.69, *MSE* = 493, $\eta_{p}^{2}$ = 0.71] versus pairs containing non-smallest numbers [12 ms; *F*_(1,26)_ = 61.79, *MSE* = 60, *p* < 0.01, $\eta_{p}^{2}$ = 0.70], replicating our previous findings (Pinhas and Tzelgov, 2012). Furthermore, as depicted in **Figure 2B**, a significant congruency × pair type × smallest number interaction, *F*_(1,26)_ = 6.99, *MSE* = 190, *p* < 0.01, $\eta_{p}^{2}$ = 0.21 demonstrated that although the modulation of the SiCE by pair type was significant for both smallest number groups [*F*_(1,14)_ = 5.28, *MSE* = 214, *p* < 0.05, $\eta_{p}^{2}$ = 0.27 and *F*_(1,12)_ = 40.44, *MSE* = 163, *p* < 0.01, $\eta_{p}^{2}$ = 0.77, for 0 and 1, respectively], it was more pronounced in the group in which 1 served as the smallest number. All other effects in the analysis were not significant.

To further investigate the sources of the interaction depicted in **Figure 2B** and to provide additional support for the idea of semantic end-value effects, a separate analysis was conducted for the group in which 0 served as the smallest number. This analysis revealed a congruency × pair type interaction, *F*_(3,45)_ = 17.15, *MSE* = 78, *p* < 0.01, $\eta_{p}^{2}$ = 0.53, demonstrating that although the SiCE was significant for each pair type (*p*s < 0.003), it was more enlarged for 0 (30 ms) and 1 pairs (34 ms) than for 2 (9 ms) and 3 pairs (11 ms), *F*_(1,15)_ = 36.27, *MSE* = 434, *p* < 0.01, $\eta_{p}^{2}$ = 0.71. Most importantly, SiCE for pairs containing the smallest number 0 did not differ from the SiCE found for pairs containing 1 when it was *not* used as an end value (*F* < 1), suggesting that 1 was automatically processed as an end value even when it was not used as such. There was also no difference between the SiCE found for 2 vs. 3 pairs (*F* < 1). Thus, it seems that the modulation of the SiCE by pair type was more pronounced for the group in which 1 served as an end value (**Figure 2B**) because for this group “non-smallest pairs” included pairs containing 2 and 3 (i.e., pairs that produce smaller SiCEs), whereas in the group in which 0 served as an end value “non-smallest pairs” also included pairs containing 1 (i.e., pairs that produce an enlarged SiCE). To complete the picture, the analysis conducted separately for the group in which 1 served as the smallest number revealed a congruency × pair type interaction, *F*_(2,30)_ = 35.5, *MSE* = 259, *p* < 0.01, $\eta_{p}^{2}$ = 0.70, demonstrating that SiCE was larger for 1 pairs (34 ms) than for 2 (2 ms) and 3 pairs (6 ms), *F*_(1,15)_ = 46.15, *MSE* = 392, *p* < 0.01, $\eta_{p}^{2}$ = 0.75. There was no significant difference between the SiCE found for 2 vs. 3 pairs (*p* > 0.17), although the effect was significant for 3 pairs (*p* < 0.002) but not for 2 pairs (*p* = 0.3).

### ERP Results

#### N1 and P2p Components

**FIGURE 3:**
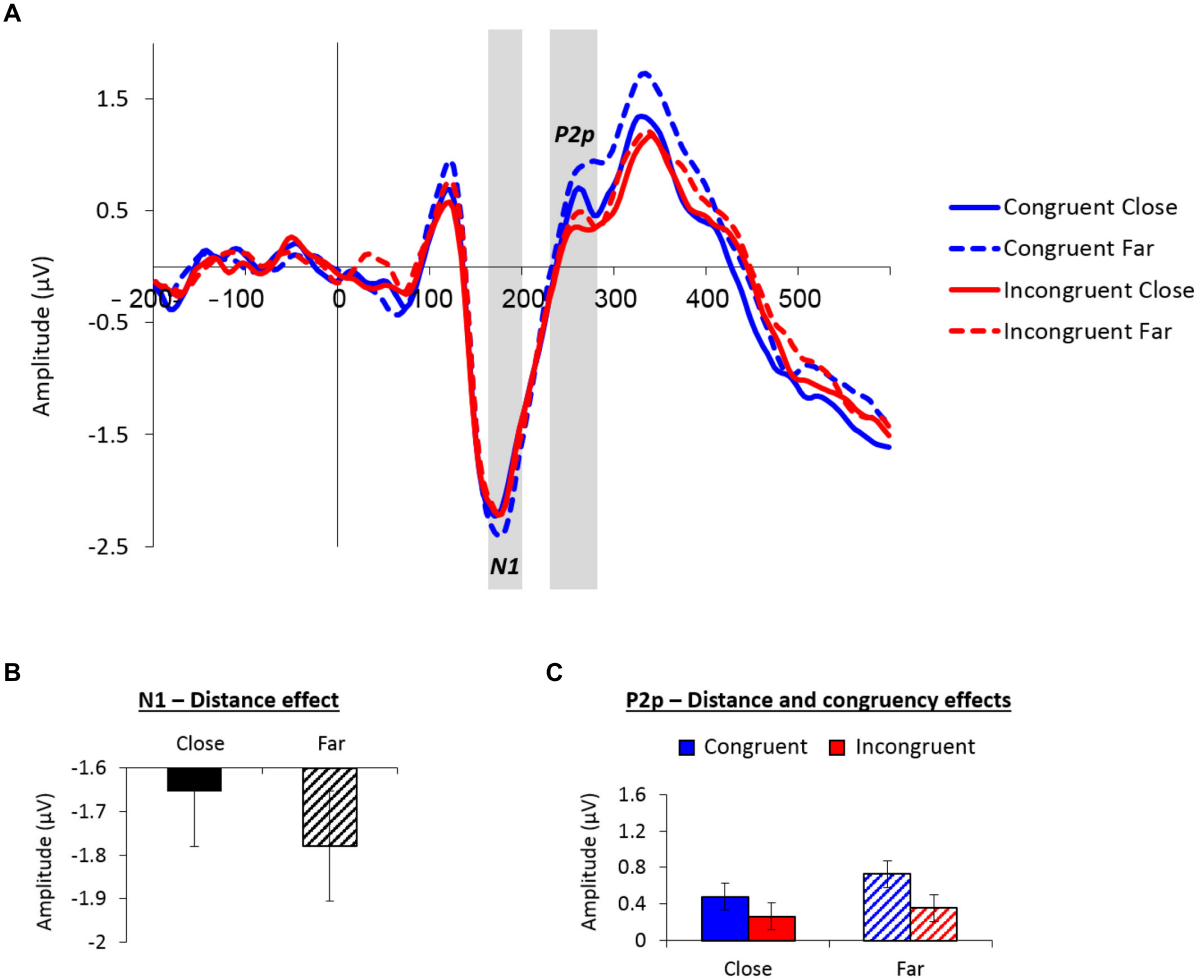
**(A)** Event-related potential (ERP) traces averaged across the nine parieto-occipital sites used for analysis as a function of congruency and distance. The N1 peaked at around 175 ms poststimulus, the P2p peaked at around 255 ms poststimulus. These components were larger for far versus close distances. **(B)** Mean amplitude values (microvolts) of the N1 (165–200 ms) as a function of distance. **(C)** Mean amplitude values (microvolts) of the P2p (230–280 ms) as a function of congruency and distance. Error bars in panels **(B)** and **(C)** denote 0.95 confidence intervals.

**Figure 3A** depicts the averaged waveform at the parieto-occipital group of electrodes as a function of congruency and distance across groups (see **Supplementary Figure S1** for the individual waveforms).

The analysis of the posterior N1 (165–200 ms) revealed a marginal main effect of distance, *F*_(1,26)_ = 4.08, *MSE* = 0.11, *p* = 0.05, $\eta_{p}^{2}$ = 0.14, indicating larger negative amplitudes for far (-1.87 μV) versus close (-1.74 μV) distances (see **Figure 3B**). There was also a marginally significant congruency × smallest number interaction, *F*_(1,26)_ = 4.19, *MSE* = 0.26, *p* = 0.05, $\eta_{p}^{2}$ = 0.14; however, *post hoc* contrasts did not reveal significant effects of congruency for any of the smallest number groups (*p*s > 0.14).

The analysis of the P2p (230–280 ms) revealed a main effect of distance, *F*_(1,26)_ = 5.88, *MSE* = 0.14, *p* < 0.05, $\eta_{p}^{2}$ = 0.18, demonstrating greater parieto-occipital positivity for far (0.54 μV) versus close (0.37 μV) distances. Furthermore, a main effect of congruency, *F*_(1,26)_ = 8.27, *MSE* = 0.29, *p* < 0.01, $\eta_{p}^{2}$ = 0.24, exhibited larger positive amplitudes for congruent (0.60 μV) versus incongruent (0.31 μV) trials. Both main effects are depicted in **Figure 3C**. No other effects in the analysis were significant.

#### P3 Component

**FIGURE 4:**
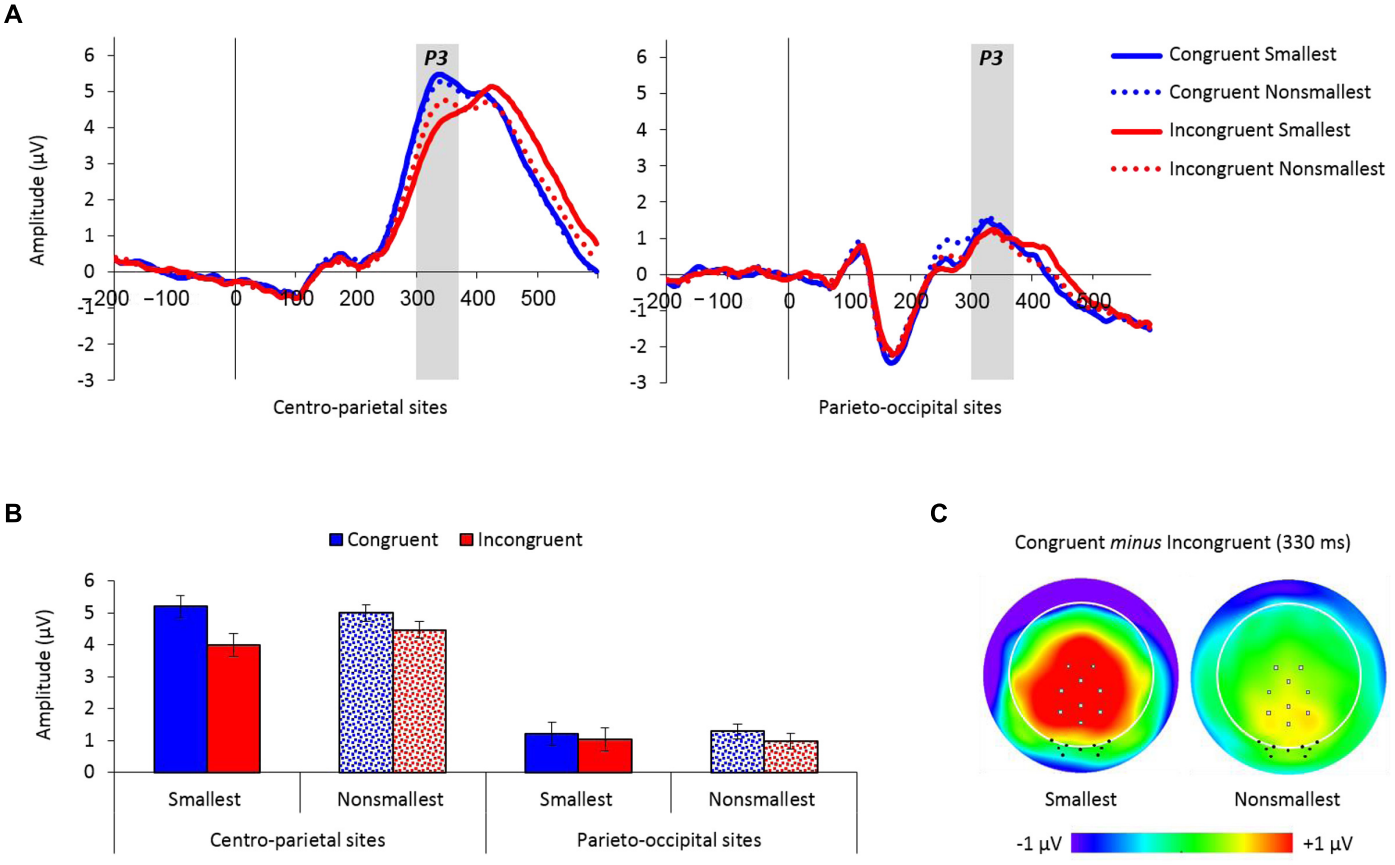
**(A)** Event-related potential traces averaged across the nine centro-parietal sites and the nine parieto-occipital sites used for analysis, as a function of congruency and pair type. The P3 peaked at around 330 ms poststimulus and was enlarged for congruent versus incongruent trials over centro-parietal sites, but not over parieto-occipital sites. **(B)** Mean amplitude values (microvolts) by congruency, pair type and electrodes location from 300 to 370 ms. Error bars denote 0.95 confidence intervals. **(C)** Topographic distributions (top view, full projection) of congruent minus incongruent trials at 330 ms for pairs containing the smallest number and for pairs containing non-smallest numbers. Centro-parietal sites are marked with gray squares and parieto-occipital sites are marked with black dots. The congruency effect (congruent minus incongruent trials) was enlarged for pairs containing the smallest number only over centro-parietal sites.

**Figure 4A** depicts the averaged waveforms for both centro-parietal and parieto-occipital groups of electrodes as a function of congruency and pair type (see **Supplementary Figures S2** and **S3** for the individual waveforms).

The mean amplitude analysis of the P3 component (300–370 ms) revealed a main effect of congruency demonstrating increased amplitudes for congruent (3.17 μV) versus incongruent (2.61 μV) trials, *F*_(1,26)_ = 47.45, *MSE* = 0.74, *p* < 0.01, $\eta_{p}^{2}$ = 0.65. Congruency was further modulated by electrodes location, *F*_(1,26)_ = 16.46, *MSE* = 0.68, *p* < 0.01, $\eta_{p}^{2}$ = 0.39, exemplifying a larger congruency effect over centro-parietal sites [5.10 vs. 4.23 μV for congruent and incongruent trials, respectively; *F*_(1,26)_ = 49.12, *MSE* = 0.87, *p* < 0.01, $\eta_{p}^{2}$ = 0.65] compared to parieto-occipital sites [1.24 vs. 0.99 μV for congruent and incongruent trials, respectively; *F*_(1,26)_ = 6.06, *MSE* = 0.54, *p* < 0.05, $\eta_{p}^{2}$ = 0.19]. Importantly, there was also a significant congruency × pair type × electrodes location interaction, *F*_(1,26)_ = 12.06, *MSE* = 0.39, *p* < 0.01, $\eta_{p}^{2}$ = 0.32, demonstrating a significant modulation of congruency by pair type over centro-parietal sites [*F*_(1,26)_ = 15.94, *MSE* = 0.41, *p* < 0.01, $\eta_{p}^{2}$ = 0.38], but not over parieto-occipital sites (*F* < 1). Follow-up comparisons conducted for the congruency × pair type interaction over centro-parietal sites showed that although the congruency effect was significant for both pair types [pairs containing the smallest number: *F*_(1,26)_ = 48.71, *MSE* = 0.85, *p* < 0.01, $\eta_{p}^{2}$ = 0.65; pairs containing non-smallest numbers: *F*_(1,26)_ = 18.45, *MSE* = 0.43, *p* < 0.01, $\eta_{p}^{2}$ = 0.42], it was enlarged for pairs containing the smallest number versus pairs containing non-smallest numbers across groups (see **Figure 4**). Two other effects in the analysis were significant but had no important theoretical implications: (a) a main effect of electrodes location, *F*_(1,26)_ = 28.71, *MSE* = 48.94, *p* < 0.01, $\eta_{p}^{2}$ = 0.52, showed increased amplitude values over centro-parietal sites (4.66 μV) than over parieto-occipital sites (1.11 μV), and (b) an interaction between the smallest number × pair type × electrodes location, *F*_(1,26)_ = 4.6, *MSE* = 0.78, *p* < 0.05, $\eta_{p}^{2}$ = 0.15, however, follow-up comparisons revealed that the modulation of the smallest number by pair type was not significant over centro-parietal sites (*p* = 0.08), as well as over parieto-occipital sites (*p* = 0.18). All other effects in the analysis were not significant.

**FIGURE 5:**
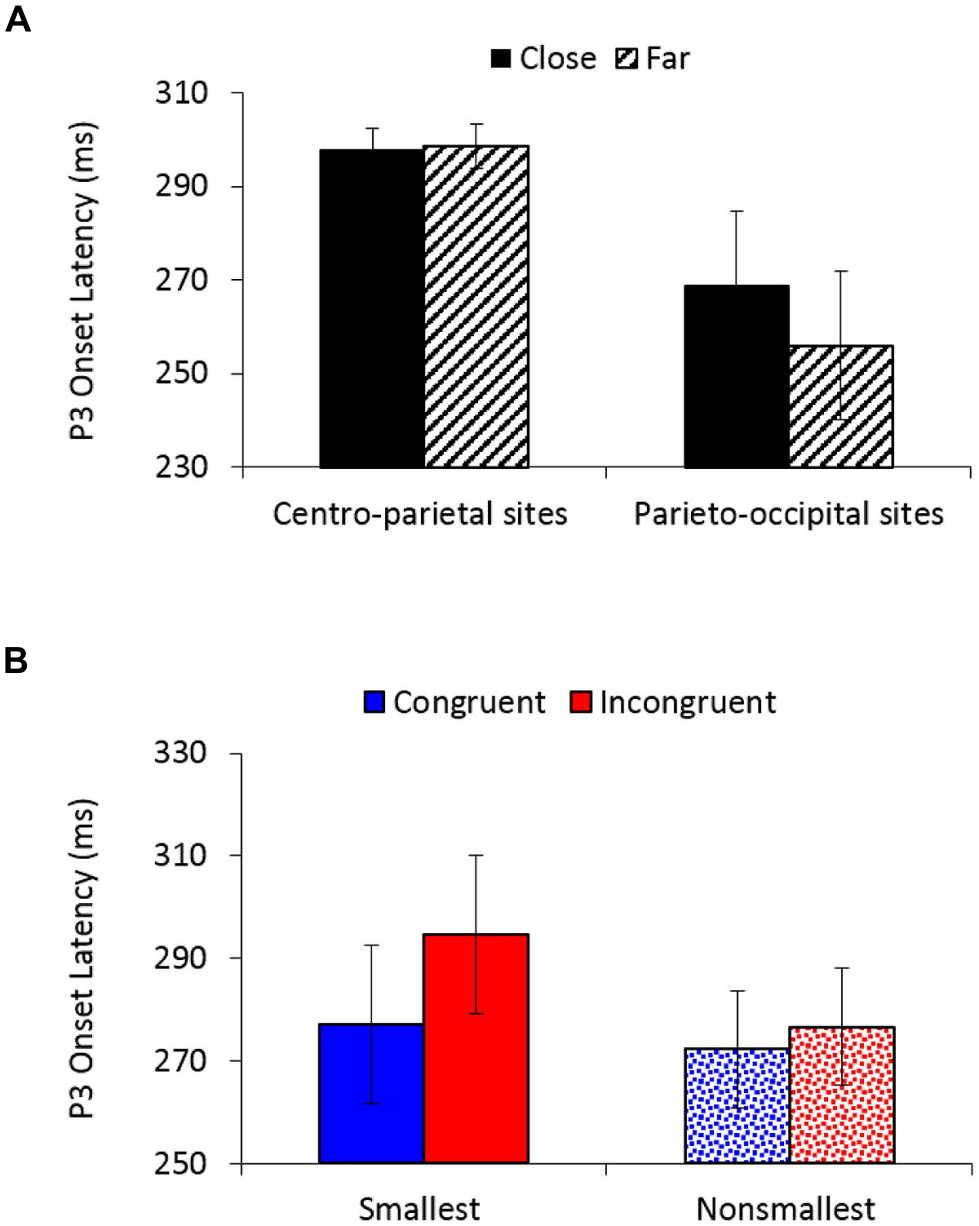
**(A)** P3 onset latency (in ms) as a function of distance and electrodes location. Error bars denote 0.95 confidence intervals. **(B)** P3 onset latency (in ms) as a function of congruency and pair type. Error bars denote 0.95 confidence intervals.

P3 onset latency analysis (250–500 ms) revealed a significantly earlier P3 onset for congruent (275 ms) than for incongruent trials (286 ms), *F*_(1,26)_ = 9.62, *MSE* = 1,383, *p* < 0.01, $\eta_{p}^{2}$ = 0.27, as well as for far (277 ms) than for close comparisons (283 ms), *F*_(1,26)_ = 5.01, *MSE* = 775, *p* < 0.05, $\eta_{p}^{2}$ = 0.16. A main effect of electrodes location, *F*_(1,26)_ = 8.41, *MSE* = 16,981, *p* < 0.01, $\eta_{p}^{2}$ = 0.24, demonstrated an earlier P3 onset over parieto-occipital sites (262 ms) than over centro-parietal sites (298 ms). A marginally main effect of pair type, *F*_(1,26)_ = 4.06, *MSE* = 3,586, *p* = 0.05, $\eta_{p}^{2}$ = 0.14, indicated an earlier P3 onset for comparisons of non-smallest numbers (274 ms) than for comparisons to the smallest number (286 ms). Distance interacted with electrodes location, *F*_(1,26)_ = 5.05, *MSE* = 1,047, *p* < 0.05, $\eta_{p}^{2}$ = 0.16 (**Figure 5A**), demonstrating significantly earlier P3 onset for far versus close comparisons over parieto-occipital sites (256 vs. 269 ms; *F*_(1,26)_ = 5.43, *MSE* = 1,677, *p* < 0.05, $\eta_{p}^{2}$ = 0.17), but not over centro-parietal sites (299 vs. 298 ms; *F* < 1, respectively). Importantly, congruency interacted with pair type, *F*_(1,26)_ = 4.39, *MSE* = 1,086, *p* < 0.05, $\eta_{p}^{2}$ = 0.14 (**Figure 5B**), indicating significantly greater delay in P3 onset for incongruent compared to congruent trials when comparing to the smallest number [295 vs. 277 ms; *F*_(1,26)_ = 10.65, *MSE* = 1,595, *p* < 0.01, $\eta_{p}^{2}$ = 0.29], but not when comparing non-smallest numbers [277 vs. 272 ms; *F*_(1,26)_ = 1.23, *MSE* = 873, *ns*,$\eta_{p}^{2}$ = 0.05, respectively). All other effects in the analysis were not significant.

### Source Localization of the End Effect

**FIGURE 6:**
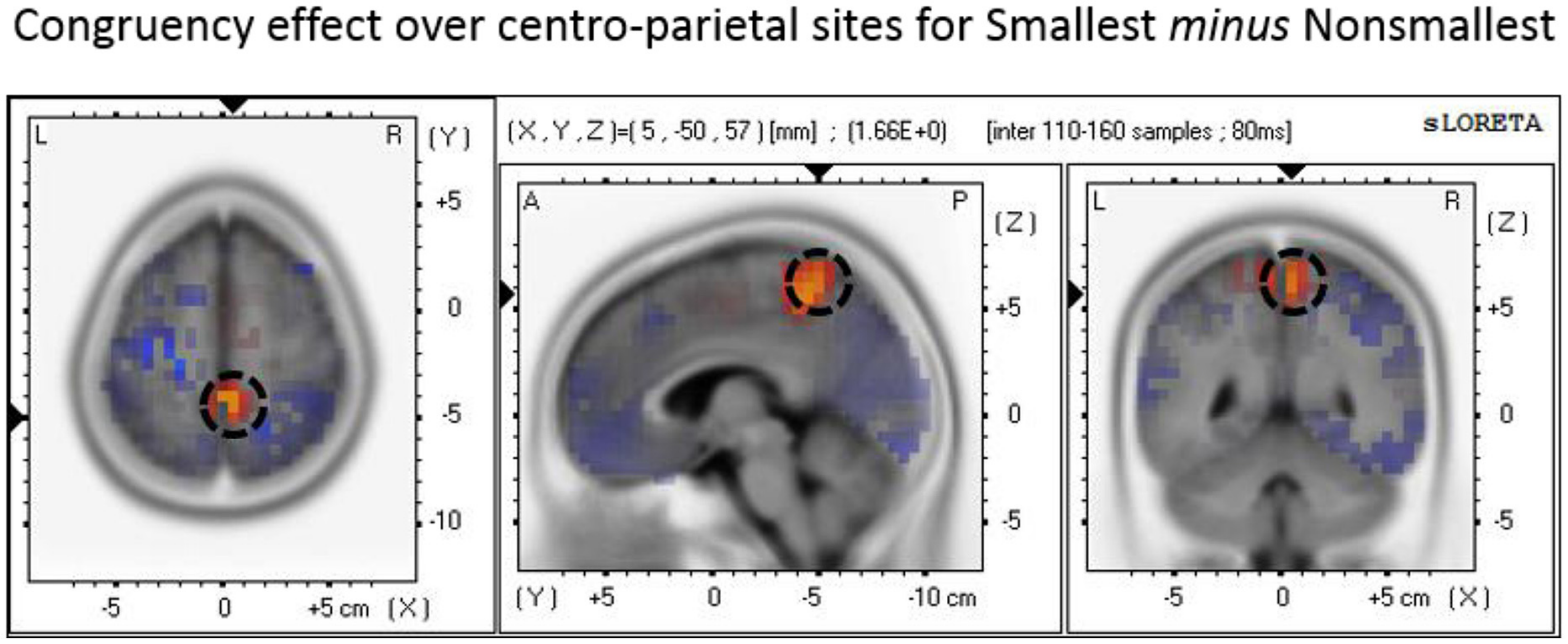
**Source localization of the P3 component over centro-parietal sites discriminating the congruency effect for smallest versus non-smallest comparisons.** The panel represents the congruency effect (congruent minus incongruent) for pairs containing the smallest number minus the congruency effect (congruent minus incongruent) for non-smallest pairs. The dashed circle marks the precuneus region where the brain activity related to the described effect was found to be maximal.

The P3 mean amplitude analysis revealed increased congruency effect (i.e., congruent minus incongruent) amplitudes for pairs containing the smallest number than for pairs containing non-smallest numbers over centro-parietal sites. To explore the neural generators of the end effect as reflected by this (simple) interaction, we conducted a source localization analysis using paired *t*-tests. The analysis indicated that the maximal difference between the congruency effect found for pairs containing the smallest number versus the congruency effect found for non-smallest pairs was localized to the precuneus (Brodmann area 7; MNI coordinates: *X* = 5, *Y* = -50, *Z* = 60; see **Figure 6**).

## Discussion

The present study explored the brain correlates and neural sources associated with processing semantic end values. For these purposes, we tested whether adult ERPs would implicate the involvement of the end-anchor identification process (Leth-Steensen and Marley, 2000; Pinhas and Tzelgov, 2012), which is an aspect of task-irrelevant numerical processing elicited during physical size comparisons. As expected, SiCE was larger for comparisons to the smallest number than for non-smallest comparisons, replicating our previous behavioral findings (Pinhas and Tzelgov, 2012). Importantly, a similar congruency-related modulation was manifested in a centro-parietal P3 ERP component, demonstrating an enhanced P3 congruity effect (congruent minus incongruent) for pairs containing the smallest number than for those containing non-smallest numbers. These differences in the P3 congruity effect were localized to the precuneus. Furthermore, there was a greater delay in P3 onset for incongruent versus congruent trials when comparing to the smallest number, but not when comparing non-smallest numbers. Thus, both behavioral and electrophysiological results reveal that participants encoded whether or not the pair contained a numerical end value. Consistently with previous studies in the relevant extant literature, we found parieto-occipital ERP distance effects. The effect was marginal for the N1 and pronounced for the P2p.

### The Behavioral SiCE as an Indicator for Semantic Lower End Values

The behavioral data replicated Pinhas and Tzelgov’s (2012) findings, which showed an enlarged SiCE for pairs containing the smallest number 0 or 1 versus the SiCE found for pairs containing non-smallest numbers. Given that 1 is much more frequent than 0 (Dehaene and Mehler, 1992), we believe that the similarities observed in responses for pairs containing the smallest number 0 or 1 cannot be explained (or predicted) by assuming a similar level of familiarity for these numbers (e.g., Verguts and Van Opstal, 2014). Instead, we propose that an enlarged SiCE for pairs containing the smallest number 0 or 1 is in line with an end-anchor identification process that leads to the observation of a behavioral automatic end effect for numbers that are assumed to be encoded in long-term semantic memory as “the smallest” based on prior real-life experience (Pinhas and Tzelgov, 2012). Additional support for the idea of a special status for 0 and 1 as end values comes from our behavioral findings of an enlarged SiCE for pairs containing 1 even when it was *not* used as the smallest number in the set (see also Pinhas and Tzelgov, 2012). In turn, this finding implies that 1 may be semantically marked as an end value when processed automatically, even if its current episodic serial position in the set is not of an end value. We are not claiming here that the ordinal relations between 0 and 1 are not realized and being acted upon when these number values are processed intentionally, that is, processed as part of the task requirements. Furthermore, a closer inspection of our behavioral data further reveals that physical size comparisons of 0 with 1 resulted in a significant SiCE^2^, suggesting that the ordinal relations between 0 and 1 are also realized when these number values are processed automatically. Instead, we are suggesting that since both 0 and 1 (in the absence of 0) are usually experienced in daily life as being “the smallest” or “the first,” they are automatically tagged as such, which in turn results in larger SiCE when they are physically compared with other numbers. In contrast, other relatively small numbers (e.g., 2, 3) do not produce the automatic end effect regardless of whether they serve as end values (Pinhas and Tzelgov, 2012).

Another aspect that should be considered as possibly contributing to the acquired special status of 0 and 1 is the widely accepted assumption that numerical discrimination in human and non-human animals is based on the ratio between the presented numerosities, in accordance with Weber’s law (e.g., Feigenson et al., 2004; Cantlon and Brannon, 2006; Halberda et al., 2012). However, discriminating 0 from other numbers based on a ratio seems implausible given that dividing by 0 is mathematically undefined (at least in elementary arithmetic) and thus does not make sense. Furthermore, while discriminating 1 based on ratio is mathematically possible, it is unique in the sense that dividing by 1 always equals the dividend (i.e., the number that is being divided by 1). Accordingly, these notable characteristics for discriminating 0 and 1 from other numbers may itself contribute to uniquely categorizing these numbers as lower end values. It should, however, be pointed out that an earlier alternative model by Dehaene (1989; see also Jamieson and Petrusic, 1975; Holyoak, 1978) suggested that numerical discrimination is not based on the ratio between the two to-be-compared numbers. Instead, it is assumed that comparison is based on the ratio of the distance between each number and a reference point located at the end of the number sequence (i.e., the smallest/largest number in the set). If performance is indeed determined by the ratio between these two distances (Dehaene, 1989), then the problems or unique characteristics associated with discrimination based on ratios involving 0 and 1 discussed earlier do not seem to apply.

### The Congruency-related P3

As expected, the event-related neural activity revealed a spatially extended centro-parieto-occipital P3 that differentiated congruent from incongruent trials, with the latter having decreased amplitudes and delayed onset latency compared to the former (e.g., Schwarz and Heinze, 1998; Cohen Kadosh et al., 2007; Szűcs and Soltész, 2007; Szűcs et al., 2007, 2009; Parnes et al., 2012). Importantly, over centro-parietal sites, the P3 congruency effect (congruent minus incongruent) was further modulated by pair type, such that pairs that contained the smallest number elicited increased congruency effect amplitude differences than pairs that contained non-smallest numbers. The same modulation of congruency by pair type was apparent in the P3 onset latency analysis across both centro-parietal and parieto-occipital electrodes, revealing a greater delay in P3 onset for incongruent vs. congruent trials in comparisons to the smallest number versus comparisons of non-smallest numbers.

The P3 component is thought to indicate stimulus evaluation and classification processes. Previous research has shown that P3 amplitude depends on target probability and that P3 latency indexes stimulus evaluation time relatively independent of response selection and/or execution processes (e.g., Kutas et al., 1977; McCarthy and Donchin, 1981; Kok, 2001; Polich, 2007, 2012). Accordingly, the presentation of incongruent pairs with the smallest number 0 or 1 (i.e., trials in which 0 or 1 are presented in a larger font size) might have elicited a greater subjective “feeling” of oddness or surprise relative to incongruent trials with non-smallest numbers, whereas the presentation of congruent trials with the smallest number 0 or 1 (i.e., trials in which 0 or 1 are presented in a smaller font size) might have elicited a greater subjective “feeling” of sensibleness relative to congruent trials with non-smallest numbers. Consequently, these subjective evaluations might have elicited a larger P3 congruity effect (congruent minus incongruent) for comparisons to the smallest number than for non-smallest numbers. Consistent with this suggested interpretation, it should take longer to evaluate “odd targets” than “sensible targets,” resulting in the observed P3 onset latency effects. Thus, both P3 amplitude and latency seem to mark the electrophysiological correlate for encoding the serial position of the smallest end value in the set, resulting in slower classification of numbers that are semantically tagged as “smallest” as being physically larger. It is also worth noticing that the congruency × pair type × smallest number interaction observed in RTs was not apparent in the P3 onset latency analysis. Instead, the latter revealed a similar modulation of congruency by pair type for both smallest number groups. This could suggest that the obtained RT differences relate to differences in response selection and/or execution processes, instead of a stimulus evaluation effect (McCarthy and Donchin, 1981). Future research is needed to determine this possibility.

Moreover, our data suggest that the maximal difference between the centro-parietal P3 congruity effect found in pairs containing the smallest number versus non-smallest pairs is localized to the precuneus. In other words, the results of the source localization analysis raise the hypothesis that the precuneus may be the brain region in which the presence of end stimuli has the stronger effect on the automatic processing of number values. Functional neuroimaging studies suggest that this medial area of the superior parietal cortex plays a central role in a wide variety of tasks such as shifting attention in visuo-spatial imagery, episodic memory retrieval, and self-processing operations (for a review see Cavanna and Trimble, 2006). Thus, it is clear that the precuneus is not specific to number processing. Nevertheless, numerous studies have reported that the precuneus is also active in number-related tasks including numerical and physical comparison (e.g., Pesenti et al., 2000; Pinel et al., 2001; Ansari et al., 2005, 2006; Kaufmann et al., 2005), approximation (Dehaene et al., 1999), calculation (e.g., Delazer et al., 2005; Ischebeck et al., 2006; Fehr et al., 2007; Arsalidou and Taylor, 2011), and counting (Piazza et al., 2002). Although there has not been much consideration in the literature regarding the role that the precuneus may play in number processing, it has been suggested that this region contributes to a covert attentional process that operates when selecting quantity locations on the mental number line (Dehaene et al., 2003). This region also reflects visual imagery processes involved in the retrieval and the execution of complex calculations (Delazer et al., 2005). Furthermore, a recent study by Krause et al. (2014) compared participants’ spatial and non-spatial responses in a parity judgment task. The authors found that inter-individual variability in the linkage between numbers and spatial responses (as instantiated by the SNARC effect; cf. Dehaene et al., 1993) correlated with variations in gray matter volume around the right precuneus, thus reflecting the involvement of this area in the spatial representation of numbers. Consistent with these suggestions, a plausible interpretation of our findings is that the process of encoding the serial position of a number as an end versus a non-end value possibly involves the precuneus because it relies on a covert shifting of attention in visuo-spatial imagery. This covert encoding process probably involves shifting one’s attention to the relevant quantity locations along the spatially oriented mental number line. As such, this process is presumably affected by the specific number range presented in the experiment and may be more efficient in locating the end values of the given number range because they are more salient and/or serve as anchors. However, given the difficulties in determining source configuration from scalp topography and assessing the accuracy of a given solution these suggestions should be taken with caution (e.g., Luck, 2014). More research is needed to validate these speculated hypotheses and to better understand the functional role of the precuneus in number processing.

### The Distance Effect in RTs and ERPs

In line with past studies, our data also indicated the involvement of an analog comparison process that contrasts task-irrelevant number values along an ordered continuum of magnitudes (Leth-Steensen and Marley, 2000; Pinhas and Tzelgov, 2012), which was manifested in both behavioral and neural distance effects. Behaviorally, the SiCE increased with the increase in the intrapair distance, replicating previous results (e.g., Henik and Tzelgov, 1982; Tzelgov et al., 1992; Pansky and Algom, 1999; Schwarz and Ischebeck, 2003; Cohen Kadosh et al., 2007; Parnes et al., 2012; Pinhas and Tzelgov, 2012). Yet, unlike Pinhas and Tzelgov’s (2012) findings, we found that the modulation of SiCE by distance was independent of pair type and smallest number condition. Thus, here we did not find an attenuated effect of distance for the SiCE obtained in pairs containing the smallest number 0 or 1. It seems, however, that this inconsistency can be easily reconciled when considering that Leth-Steensen and Marley’s (2000) race model predicts distance effects for end-value pairs in cases where the analog comparison process finishes before the end-value identification process. Alternatively, the fact that there was no attenuated effect of distance for the SiCE involving end value pairs might be what is expected to occur when number values are being compared *automatically* because in such cases the actual responses are determined by physical size (and not by numerical magnitude) information. Hence, once initiated, both numerical processes (i.e., the end-anchor identification process and the analog comparison process) will continue running simultaneously in the background (and contribute implicitly to the response process) until the physical comparison response is made. In contrast, when explicitly comparing number values, the numerical comparison is stopped whenever the result of either the end-anchor identification process or the analog comparison process allows for a response. This in turn may lead to attenuated distance effects for end value pairs if the end-anchor identification process runs faster (Leth-Steensen and Marley, 2000).

At the ERP level, a parieto-occipital P2p (230–280 ms) distance effect was reflected in an increased amplitude for far as compared to close numbers. The distance effect was marginally significant earlier in the waveform for the N1 (165–200 ms), whereas later in the waveform it was evident by an earlier P3 onset latency for far versus close numbers. We found no amplitude differences as a function of distance for the P3. Parietal ERP distance effects were previously reported in a variety of numerical tasks (e.g., numerical comparisons, physical comparisons) at both early and late time windows and across different number notations (e.g., Dehaene, 1996; Temple and Posner, 1998; Libertus et al., 2007; Szűcs and Soltész, 2007; Szűcs et al., 2007; Hyde and Spelke, 2009, 2012). Furthermore, the distribution of the observed distance effects is consistent with the neuroimaging and neuropsychological literature implicating the IPS in number processing (e.g., Pinel et al., 2001; Dehaene et al., 2003, 2004; Feigenson et al., 2004; Ansari et al., 2005; Kaufmann et al., 2005; Cohen Kadosh et al., 2007). Our findings of posterior ERP distance effects in a task in which participants were explicitly requested to ignore number values replicate past research and are consistent with the notion that the same mechanism of number processing is accessed for both intentional and automatic number comparison tasks. Nevertheless, Szűcs et al. (2007) found differences in the strength of early parietal distance effects observed for the same participants when they performed numerical versus physical size comparisons. Specifically, weaker distance effects were reported early in the waveform in the physical size comparison task (i.e., when number values were task-irrelevant) compared to the numerical comparison task (i.e., when number values were task-relevant). This pattern is consistent with the present findings of rather small early ERP distance effects both in terms of effect size measures and amplitude differences. This is presumably related to the automatic processing of number values.

### ERP Evidence for Two Processes Underlying Automatic Number Comparisons

More generally, our results provide novel neural evidence and replicate previous behavioral findings supporting a distinct process that identifies whether the pair includes an end value when numerical information is task-irrelevant (Leth-Steensen and Marley, 2000; Pinhas and Tzelgov, 2012). In addition, the data replicate previous studies that have repeatedly found that the numerical difference between number values is processed both at the behavioral and neural levels, even when numbers are processed automatically, thus supporting an analog comparison process. The fact that each of these processes was expressed in different ERP component(s) with different topographies supports the notion that they are indeed distinct. Furthermore, additional evidence in our study supporting the existence of two distinct processes is that the analog comparison process and the end-anchor identification process also differed in terms of their timing. The distance effect appeared earlier in the waveform (as soon as 165 ms after stimulus onset), whereas the congruency by pair type interaction appeared only at about 300 ms after stimulus onset, suggesting that the analog comparison process begins earlier than the end-anchor identification process. Yet, there was also a partial temporal overlap between these two effects as each of them modulated P3 onset latency. This temporal overlap is consistent with the idea that once the end-anchor identification process starts, it runs in parallel to the analog comparison process. In turn, this agrees with (or at least does not contradict) the hypothesis of a race model suggested by Leth-Steensen and Marley (2000). Furthermore, the results of our source localization analysis raise the question of whether the end-anchor identification process activates a different brain region (i.e., the precuneus) than the IPS, which is the area that has been typically associated with the analog comparison process (Pinel et al., 2001; Dehaene et al., 2003; Feigenson et al., 2004; Ansari et al., 2005; Kaufmann et al., 2005; Hyde and Spelke, 2012).

## Conclusion

In sum, our data suggests that the analog comparison process may be related to bottom-up or stimulus driven automatic processing of number values. The early onset of the ERP distance effects coincides with number models that assume that the representation of numbers as quantities (or the mental number line) is immediately accessed whenever numerical information is perceived (e.g., Dehaene et al., 2003; Feigenson et al., 2004). This immediate access allows the understanding of where a given quantity falls with respect to other quantities as reflected in the analog comparison process. In contrast, the end-anchor identification process may be related to the top-down visuo-spatial framing or constructing of the specific number range that is being dealt with during the task (see also Pinhas et al., 2013). This process was manifested as a modulation of the P3 congruency effect, thus it may be related to higher-order stimuli categorization and evaluation processes thought to be reflected in the P3 (e.g., Kutas et al., 1977; McCarthy and Donchin, 1981; Kok, 2001; Polich, 2007, 2012). Future work is needed to better understand how the end-anchor identification process develops in relation to the analog comparison process (Goldman et al., 2013) as well as characterizing the processing of less salient or prototypical end values by examining upper end values. We conclude that two processes are involved in the processing of numbers: an analog comparison process and an end-anchor identification process. Since both processes were indicated under conditions of automatic processing they both seem to encode the fundamental aspects of symbolic numerical information, namely, quantity information, as well as information about the range of these quantities.

## Conflict of Interest Statement

The authors declare that the research was conducted in the absence of any commercial or financial relationships that could be construed as a potential conflict of interest.

## Footnotes

1 Pinhas and Tzelgov’s (2012) stimulus set included positive as well as negative numbers. However, given that negative numbers are processed in terms of their absolute values in the physical comparison task (i.e., the minus signs attached to negative numbers are ignored) 0, 1, and 2 were perceived as the smallest numbers used for the three different group of participants.

2 *F*_(1,15)_ = 6.31, *MSE* = 172.08, *p* = 0.02, $\eta_{p}^{2}$ = 0.30.
