# Supplementary material for: The Neural Signatures of Processing Semantic End Values in Automatic Number Comparisons
Source: Front Hum Neurosci. 2015 Nov 27;9:645. doi: 10.3389/fnhum.2015.00645 (PMC4661242; doi:10.3389/fnhum.2015.00645)
Supplement: Supplementary file 1 [file Data_Sheet_1.DOCX]

**Supplementary material**

Table S1: Number pairs used in the experiment.

| *Pair Type* | *Pair* | | *Intrapair*  *Distance* |
| --- | --- | --- | --- |
|  | *Number1* | *Number2* |  |
| 0 pairs^*^ |  |  |  |
|  | 0 | 1 | close |
|  | 0 | 2 | close |
|  | 0 | 4 | far |
|  | 0 | 6 | far |
| 1 pairs^**^ |  |  |  |
|  | 1 | 2 | close |
|  | 1 | 3 | close |
|  | 1 | 5 | far |
|  | 1 | 7 | far |
| 2 pairs^**^ |  |  |  |
|  | 2 | 3 | close |
|  | 2 | 4 | close |
|  | 2 | 6 | far |
|  | 2 | 8 | far |
| 3 pairs^**^ |  |  |  |
|  | 3 | 4 | close |
|  | 3 | 5 | close |
|  | 3 | 7 | far |
|  | 3 | 9 | far |

*Note*. Each pair was presented 120 times (60 trials in the congruent condition and 60 trials in the incongruent condition). Each number appeared once on the left and once on the right side of the computer screen at each congruency condition. ^*^ = Presented only to the group in which 0 was used as the smallest number in the set. ^**^ = Presented to both groups.

*
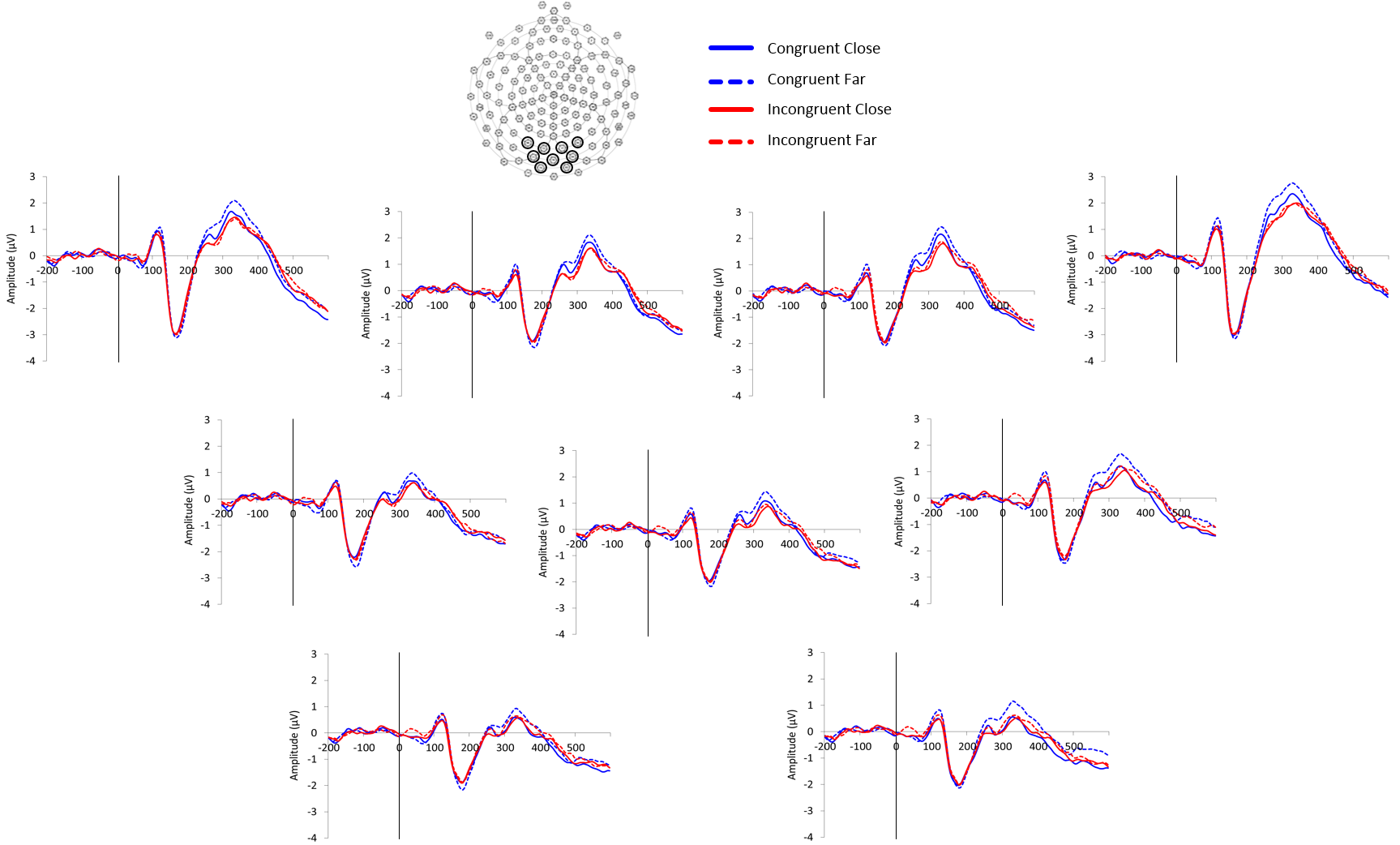
*

*Figure S1*. ERP traces at the 9 parieto-occipital sites used for the N1 and P2p analyses as a function of congruency and distance.

*
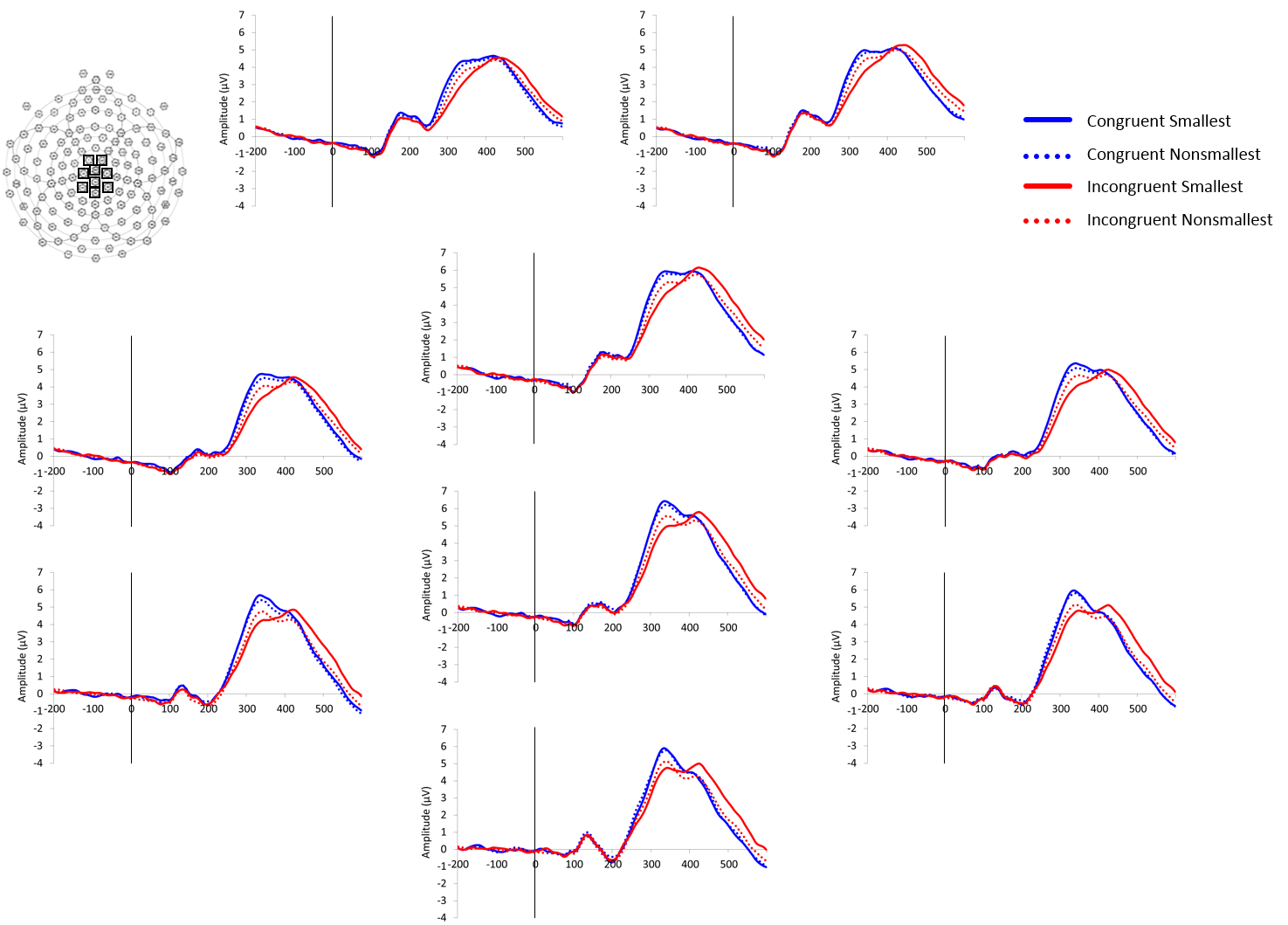
*

*Figure S2*. ERP traces at the 9 centro-parietal sites used for the P3 analysis as a function of congruency and pair type.

*
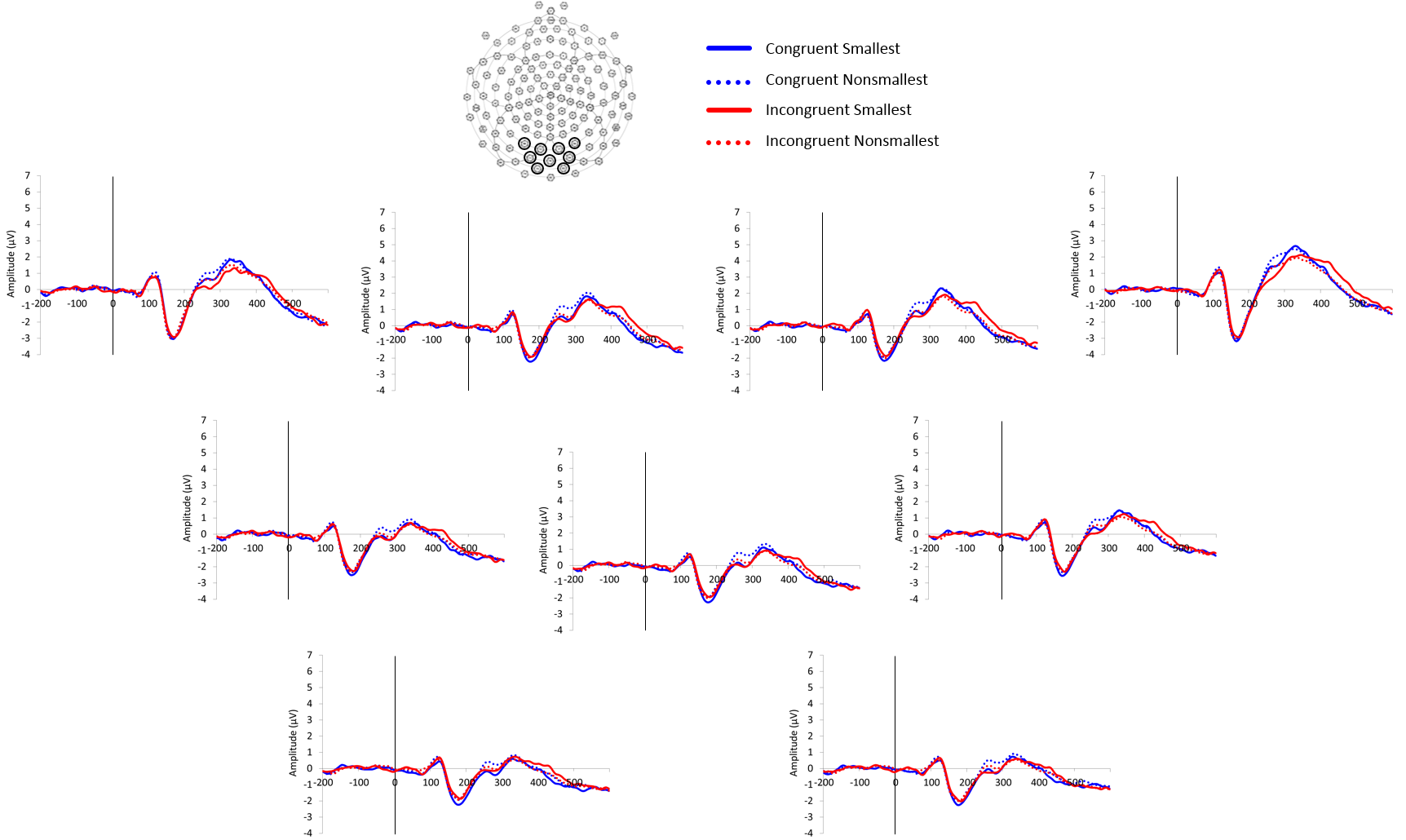
*

*Figure S3*. ERP traces at the 9 parieto-occipital sites used for the P3 analysis as a function of congruency and pair type.
